# Ramulus Mori (Sangzhi) Alkaloids (SZ-A) Ameliorate Glucose Metabolism Accompanied by the Modulation of Gut Microbiota and Ileal Inflammatory Damage in Type 2 Diabetic KKAy Mice

**DOI:** 10.3389/fphar.2021.642400

**Published:** 2021-04-15

**Authors:** Quan Liu, Shuainan Liu, Hui Cao, Wenming Ji, Caina Li, Yi Huan, Lei Lei, Yaxin Fu, Xuefeng Gao, Yuling Liu, Zhufang Shen

**Affiliations:** ^1^ Institute of Materia Medica, Chinese Academy of Medical Sciences and Peking Union Medical College, Beijing, China; ^2^ State Key Laboratory of Bioactive Substances and Functions of Natural Medicines, Key Laboratory of Polymorphic Drugs of Beijing, Institute of Materia Medica, Chinese Academy of Medical Sciences and Peking Union Medical College, Beijing, China; ^3^ Diabetes Research Center of Chinese Academy of Medical Sciences and Peking Union Medical College, Beijing, China; ^4^ Drug Delivery Technology and Novel Formulation, Institute of Materia Medica, Chinese Academy of Medical Sciences and Peking Union Medical College, Beijing, China

**Keywords:** ramulus mori (sangzhi) alkaloids, type 2 diabetes, gut microbiome, ileal damage, inflammation

## Abstract

The novel Traditional Chinese Medicine Ramulus Mori (Sangzhi) alkaloid tablets (SZ-A) are approved by The China National Medical Products Administration for the treatment of type 2 diabetes mellitus (T2DM). However, the extensive pharmacological characteristics and the underlying mechanism are unknown. This study investigated the mechanisms by which SZ-A ameliorates glucose metabolism in KKAy mice, an animal model of T2DM. Diabetic KKAy mice were treated intragastrically with SZ-A once daily for 8 weeks, after which glucose levels, lipid metabolism, gut microbiome, systemic inflammatory factors, luminal concentrations of short-chain fatty acids (fecal samples), and ileal proteomic changes were evaluated. The ileum tissues were collected, and the effects of SZ-A on pathological inflammatory damage were evaluated by hematoxylin and eosin staining, immunofluorescence, and immunohistochemistry. The mRNA and protein expression levels of various inflammatory markers, including monocyte chemoattractant protein-1 and phosphorylated nuclear factor kappa B p65, were detected in the ileum tissues. SZ-A improved glucose metabolism with enhanced insulin response and elevated glucagon-like peptide 1 (GLP-1) nearly 2.7-fold during the glucose tolerance test in diabetic KKAy mice. Gut microbiota analysis demonstrated that SZ-A administration elevated the abundance of *Bacteroidaceae* and *Verrucomicrobia*, reduced the levels of *Rikenellaceae* and *Desulfovibrionaceae;* and increased the concentrations of fecal acetic and propionic acids compared to the diabetic model group. Additionally, SZ-A markedly improved ileal inflammatory injury and pro-inflammatory macrophage infiltration and improved intestinal mucosal barrier function in diabetic KKAy mice. SZ-A also attenuated the levels of circulating endotoxin, pro-inflammatory cytokines, and chemokines in the mice sera. Collectively, SZ-A ameliorated the overall metabolic profile including glucose and lipid metabolism in KKAy mice, which may be associated with an improvement in GLP-1 and insulin secretion, at least in part by modulating the gut microbiome and relieving the degree of ileal and systemic inflammation.

## Introduction

The gut microbiome plays important roles in the regulation of glucose and energy homeostasis. It also plays a critical role in obesity, glycemic control, and type 2 diabetes mellitus (T2DM) (Harris et al., 2012), which is a chronic and multifactorial disease in which diverse physiopathologic mechanisms lead to a persistent state of hyperglycemia. T2DM is fundamentally the result of beta cell and alpha cell dysfunction, and insulin resistance in different tissues of the body (Kahn et al., 2014). T2DM may also be due to the activation of pro-inflammatory mechanisms that involve several factors. Gut microbiota-mediated low-grade inflammation is also involved in the onset and progression of T2DM. Studies in mice and humans have shown that there is dysregulation in the gut microenvironment accompanied by immunological and metabolic dysfunctions in individuals who have T2DM (Ge et al., 2018).

Metabolites derived from the gut microbiota, such as short-chain fatty acids (SCFAs) and lipopolysaccharides (LPS), may act as potent immune modulators (Blaak et al., 2020). During eubiosis, the production of SCFAs is essential for maintaining the integrity of the intestinal barrier as well as for immunogenic tolerance. In addition, the effects of SCFAs are not limited to immunomodulatory functions (Salazar et al., 2020), as they can also stimulate the secretion of intestinal peptides that participate in the regulation of appetite and insulin secretion such as glucagon-like peptide 1 (GLP-1) (Grasset et al., 2017). Conversely, in the presence of gut dysbiosis during the progression of T2DM, diet-driven unfavorable microbiota composition can lead to the increased production of pro-inflammatory LPS, which are associated with alterations in gut permeability (Fuke et al., 2019). Subsequently, these inflammatory states might exacerbate the disruption of the mucus layer barrier and increase the epithelial permeability of the small intestine, resulting in elevated LPS levels in the bloodstream, metabolic endotoxemia (Kuti et al., 2020), increased levels of systemic inflammatory mediators, adiposity, obesity, insulin resistance, and hyperglycemia.

Thus, restoration of gut dysbiosis could potentially treat metabolic disorder. Modulation of the intestinal microbiota by interventions has led to a major impact on both the immunological and metabolic functions of the host. In recent years, there has been increasing interest in investigating the use of prebiotics (non-digestible carbohydrates), probiotics (life bacteria), and anti-diabetic drugs for the modulation of gut dysbiosis (Bauer et al., 2018; Hamada et al., 2020). Traditional Chinese Medicine (TCM) has been used to manage T2DM. A large number of studies have shown that the effects of TCM may be, at least in part, *via* modulation of gut microbiota (Shao et al., 2020; Xiao et al., 2020; Zheng et al., 2020).

The novel TCM *Ramulus mori* (Sangzhi) alkaloid (SZ-A) tablets, also known as Sangzhi Zong Shengwujian, is approved by The China National Medical Products Administration (NMPA, formerly known as the China Food and Drug Administration) for the treatment of patients with T2DM (Approve Number Z20200002). The main components of SZ-A powder (materials for SZ-A tablets) include alkaloids, flavonoids, polysaccharides, coumarin, quercetin, resveratrol, amino acids, and organic acids. SZ-A is a group of effective polyhydroxy alkaloids (50% or more by weight) that potently inhibit α-glucosidase, including 1-deoxyno-jirimycin (1-DNJ), fagomine (FAG), 1,4-dideoxy-1, 4-iminod-D-arabinitol (DAB), and other soluble polyhydroxy alkaloids or glycosides with a similar structure ( Liu et al., 2019a ). In preclinical pharmacological studies, chronic treatment of SZ-A was shown to lower fasting and postprandial blood glucose levels in alloxan-induced diabetic mice and rats. SZ-A has also been shown to reduce the peak of postprandial blood glucose in sucrose/starch loading tests in both healthy and diabetic mice after a single dose through the inhibition of intestinal disaccharidases. Moreover, available evidence from our previous study suggests that SZ-A improves dyslipidemia and glucose-stimulated insulin secretion in high-fat diet-induced obese C57 mice after long-term intragastrical administration (Liu et al., 2019b). These data suggest that the beneficial role of SZ-A may involve multiple mechanisms in addition to α-glucosidase inhibition. Previous studies have systematically investigated the tissue distribution of the three major active alkaloids, and they found that 1-DNJ, FAG, and DAB are mainly found in the gastrointestinal tract, liver, and kidney, respectively (Yang et al., 2017). Therefore, we hypothesized that the anti-diabetic effects of SZ-A may be *via* regulation of gut microbiota and intestinal metabolites.

In this study, we evaluated the anti-diabetic effects and underlying mechanisms of SZ-A, especially on modulation of gut microbiota, intestinal metabolites, and gut barrier integrity in type 2 diabetic KKAy mice.

## Materials and Methods

### Reagents

SZ-A powder (lot number: 201707008, The total polyhydroxy alkaloid content in SZ-A powder is about 63% by weight, which was mainly composed of 39% of DNJ, 10.5% of FAG, and 7% of DAB), was kindly provided by the Department of Research & Development of Beijing Wehand-bio Pharmaceutical Co Ltd.

### Animal Experimental Design

Animal experiments were performed following the “3R” principles and guidelines for laboratory animals (GB14925-2001 and MOST 2006a) established by the People's Republic of China. The animal protocol used was approved by the Institutional Animal Care and Use Committee of Institute of Materia Medica (Chinese Academy of Medical Sciences and Peking Union Medical College, Beijing, China). 14-week-old male KKAy mice (30 g) were purchased from Beijing Huafukang Bioscience Co., Ltd (Beijing, China). Animals were maintained at 22 ± 2°C with a 12 h light-dark cycle with free access to food and water. The 14-week-old male KKAy mice were fed with high-fat diets (45% of energy from fat; D12451; Research Diets, United States). And after 4 weeks of high-fat diets feeding, twenty-four KKAy mice were selected and randomly divided into three groups (*n* = 8) according to the levels of blood glucose, triglyceride, total cholesterol, body weight, and percentage of blood glucose increase at 30 min after oral glucose loading: diabetic model group (DM), SZ-A-low dose-treated group (SZ-A 100, 100 mg/kg), SZ-A-high dose-treated group (SZ-A 200, 200 mg/kg). All mice were treated intragastrically with SZ-A solution or an equivalent volume of water once daily for 8 weeks. After 56 days of treatments, all of the mice were fasted overnight and weighted, then were sacrificed *via* cervical dislocation. Subsequently, the ileum tissues were isolated, fixed in paraformaldehyde solution.

### Blood Glucose, Lipid, and Glycated Hemoglobin Measurements

After 4 weeks of treatment, blood samples (10 μl from each mouse) were collected from tail tips at the baseline and 4 h after fasting. Fasting blood glucose (FBG) and postprandial blood glucose (PBG) levels were measured using the glucose oxidase method (Biosino Bio-Technology and Science Inc., Beijing, China). After 42 days of treatment, all of the mice were fasted for 4 hours with free access to water. Blood samples (20 μl from each mouse) were collected from tail tips. Blood triglycerides, total cholesterol levels, and glycated hemoglobin (HbA1c) levels were assessed using commercial kits (A5911; Homa Biological, Beijing, China).

### Oral Glucose-Stimulated Insulin and Glucagon-like Peptide 1 (GLP-1) Secretion Test

To evaluate the response of insulin and GLP-1 secretion after the oral glucose stimulation, oral glucose-stimulated insulin and GLP-1 secretion test were performed after 5 weeks of treatment. All of the 24 mice were fasted overnight and given d-glucose (2 g/kg) intragastrically, and the orbital blood samples were collected at 0 and 15 min after glucose administration. The levels of insulin and active GLP-1 in blood were monitored using ELISA kits (80-INSMSU-E10; 43-GP1HU-E01; ALPCO, United States).

### Gut Microbiota Profiling and Fecal Short-Chain Fatty Acids Analysis

All of the 24 mice were sacrificed and the luminal contents were collected from the ileum (as the fecal samples) and snap-frozen in liquid nitrogen after 40 days at the end of treatment, followed by storage at −80°C. The gut microbiome in feces was assayed and the abundance and diversity of gut microbiota were analyzed using Illumina MiSeq sequencing (Major Bio-Pharm Technology, Shanghai, China) according to the standard protocol as previously described (Li et al., 2020). The sequence data were processed and analyzed on the free online Majorbio I-Sanger Cloud Platform (www.i-sanger.com). SCFAs in these fecal samples were detected based on our previous report (Cao et al., 2020). Briefly, the SCFAs in each sample were assayed by gas chromatography coupled to a mass spectrometer detector (GC-MS) (Agilent Technologies Inc. CA, United States) and quantified using Masshunter quantitative software. Correlation analysis of SCFAs and gut microbiota was performed on the platform of Majorbio I-Sanger Cloud (www.i-sanger.com). R and *p* values were obtained using Spearman's rank correlation.

### Histopathological Evaluation, Immunofluorescence, and Immunohistochemistry Assay of the Ileum

About 4 cm of ileum was fixed in 4% paraformaldehyde to prepare 5 μm paraffin slides. The ileum sections from the 24 mice at the end of experiment were stained with hematoxylin and eosin (H&E) for the analysis of inflammatory changes (*n* = 8). Histopathological assessment of inflammatory and crypt damages was assessed as previously stated by a light microscope (Dieleman et al., 1998). Six randomly selected fields from each slides were analyzed. The ileum sections were also stained with the first antibodies against F4/80 (ARG22476) and CD11c (*n* = 5) (ARG59698; Arigo Biolaboratories Corp, Taiwan). For immunohistochemistry analysis, we used Anti-NF-κB p65 (phospho S536) (ab86299, Abcam, Cambridge, United Kingdom) (*n* = 5). Images were captured with a Mirax scanner (3DHISTECH, Hungary), and the area of positive points was calculated with Image Pro (MediaCybernetics, Rockville, MD).

### Cytokines and Chemokines Assay in Serum

Blood was collected when the mice were sacrificed after 40 days at the end of treatment. Serum was prepared followed by centrifuged at 4000 rpm and stored at −80°C. The concentration of Endotoxin was determined by ELISA kit, and the concentrations of Interleukin 1β (IL-1β), Interleukin 5 (IL-5), Interleukin 10 (IL-10), Interleukin 13 (IL-13), Interleukin 1a (IL-1a), Interleukin 6 (IL-6), Interleukin 12b (IL-12b), Chemokine C-C-Motif Ligand 1 (Ccl11), Chemokine C-C-Motif Ligand 4 (Ccl4), Chemokine C-C-Motif Ligand 5 (Ccl5) and CXC chemokine ligand 1 (CXCl1) in serum were determined by Luminex liquid suspension chip detection, which was performed by Wayen Biotechnologies (Shanghai, China). The mouse 23-plex Multi-Analyte kit (Bio-Plex suspension Array System; Bio-rad, Hercules, CA, United States) was used following the manufacturer's instructions. The exact protocol was administered according to what had been reported before (Wei et al., 2015).

### Western Blotting and Quantitative Real-Time Polymerase Chain Reaction

Ileum tissues were homogenized and lyzed in radio-immunoprecipitation assay buffer, and protein concentrations were determined using a BCA protein quantitation kit. Information of the antibodies are as follow, anti-CD11c (97,585, CST, United States), anti-MHC-II (68,258, CST, United States), anti-MCP1 (ARG56590, Arigo Biolaboratories Corp, Taiwan), anti-SLC5A8 (21433-1-AP), anti-MCT1 (20139-1-AP), and anti-CD36 (18836-1-AP) were from Proteintech Group Inc. Zonula Occludens-1 (ZO-1, 61-7300, Invitrogen, United States), β-Actin antibody (C1313) and secondary antibodies were from (Applygen Technologies Inc., China). Protein levels were normalized to those of β-actin. Quantitative real-time PCR was conducted as previously described. The detailed procedure is presented in the Supplementary Methods. The primer sequences used in this study are shown in Supplementary Table S1.

### Tandem Mass Tagging Proteomics Analysis

The primary experimental procedures for TMT proteomics analysis include protein preparation, trypsin digestion, TMT labeling, HPLC fractionation, LC-MS/MS analysis, and data analysis. The detailed procedure is presented in the Supplementary Material. The TMT proteomics analysis in our research is supported by Jingjie PTM BioLabs.

### Statistical Analysis

The data are presented as the mean ± SEM. Statistical analysis was performed using GraphPad Prism 7.0. Differences in FBG, PBG, insulin, GLP-1, lipid levels, and body weight were assessed using a two-way analysis of variance (ANOVA) with Tukey’s test. Data sets involved in two groups or multiple groups were analyzed using unpaired two-tailed Student's t-test or one-way ANOVA depending on the experiments. Differences with *p* < 0.05 were considered statistically significant.

## Results

### SZ-A Ameliorates Glucose Metabolism, Enhances the Insulin Response, and Elevates Active GLP-1 Levels During Oral Glucose Tolerance Tests in Diabetic KKAy Mice

**FIGURE 1:**
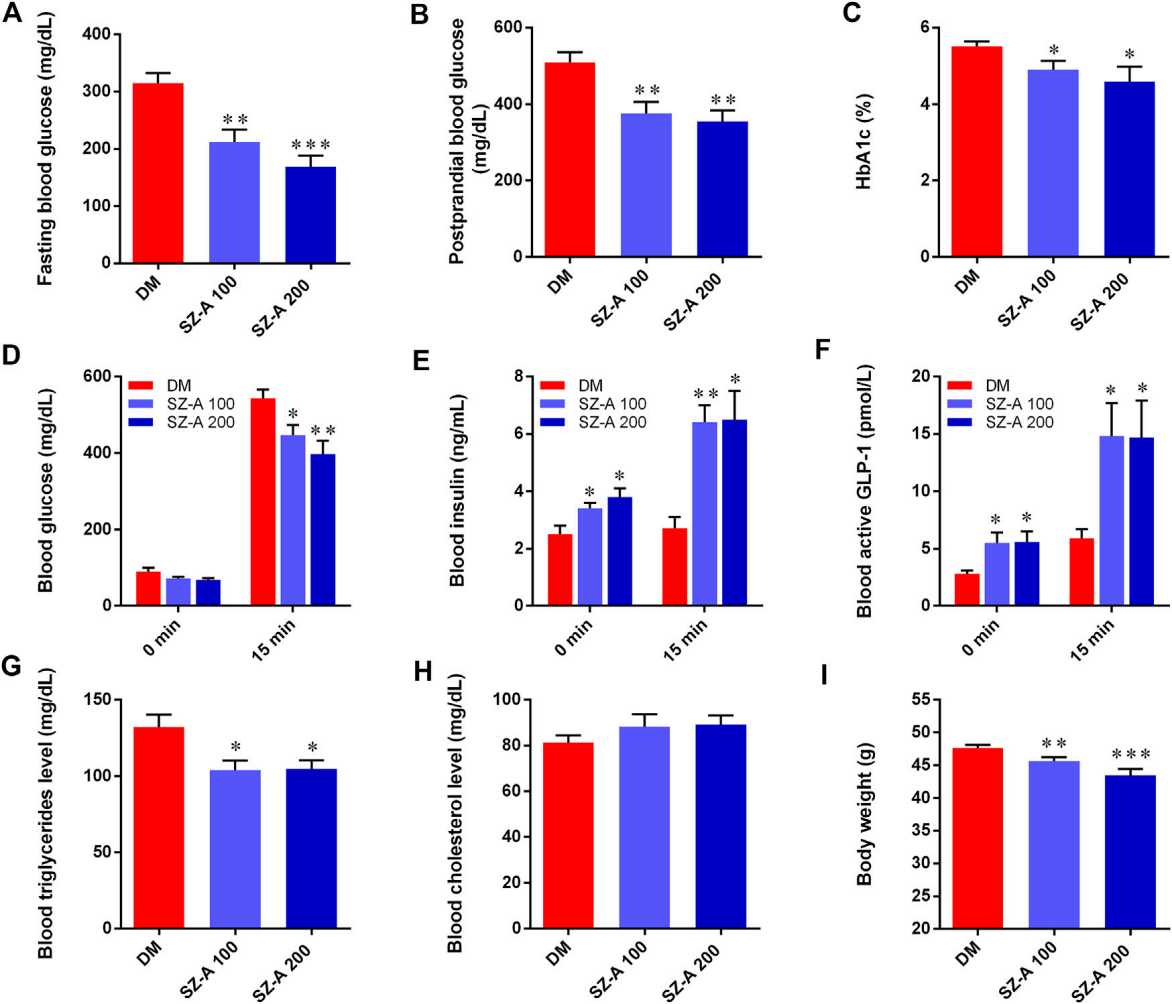
SZ-A ameliorates glucose and lipid metabolism in KKAy mice. **(A)** Fasting blood glucose. **(B)** Postprandial blood glucose. **(C)** Hemoglobin A1c (HbA1c) levels. **(D)** Blood glucose, **(E)** Blood insulin and **(F)** active glucagon-like peptide 1(GLP-1) levels at 0 and 30 min in oral glucose-stimulated insulin and GLP-1 secretion test. **(G)** Blood triglyceride levels. **(H)** Blood total cholesterol levels. **(I)** Body weight of mice. Data are expressed as mean ± standard error of the mean (SEM), *n* = 8. *p < 0.05, **p < 0.01, ***p < 0.001 *vs*. DM. DM, diabetic model group, SZ-A 100, SZ-A low-dose-treated group, SZ-A 200, SZ-A-high-dose-treated group.

After a 4-week treatment, the levels of fasting blood glucose (*p* < 0.01, *p* < 0.001) and postprandial blood glucose (*p* < 0.01, *p* < 0.01) in both SZ-A-treated groups were significantly decreased compared to the DM group (Figures 1A,B). Hemoglobin A1c (HbA1c) levels in SZ-A-treated groups were lower than those in the DM group after a 6-weeks treatment (*p* < 0.05, *p* < 0.05; Figure 1C), indicating that SZ-A exhibited good glycemic control in the KKAy mice during chronic treatment. As shown in Figure 1D, compared to the DM group, both doses of SZ-A significantly reduced blood glucose levels at 15 min after oral glucose loading (*p* < 0.05, *p* < 0.01). We further detected the blood insulin content and active GLP-1 levels as an indication of insulin and GLP-1 secretory function, respectively. As shown in Figures 1E,F, there was no notable increase in blood insulin content and active GLP-1 level in the DM group after oral glucose loading; however, SZ-A-treated groups had increased both insulin content and active GLP-1 levels at both baseline and 15 min after glucose stimulation. SZ-A 100 and SZ-A 200 significantly enhanced insulin secretion nearly 1.73-fold and 1.88-fold from baseline at 15 min after glucose stimulation, respectively (*p* < 0.01, *p* < 0.05), compared to the DM group (1.11-fold). In addition, both doses of SZ-A elevated active GLP-1 levels nearly 2.7-fold and 2.6-fold at 15 min after glucose stimulation from baseline (*p* < 0.01, *p* < 0.05), respectively, compared to the DM group (2.1-fold). Moreover, both doses of SZ-A resulted in decreased blood triglyceride levels after 6 weeks (*p* < 0.05, *p* < 0.05), and induced significant weight loss compared to the DM group at the end of treatment (*p* < 0.01, *p* < 0.001).

### SZ-A Modulates Gut Microbiota Profiling and SCFA Concentration in Feces

**FIGURE 2:**
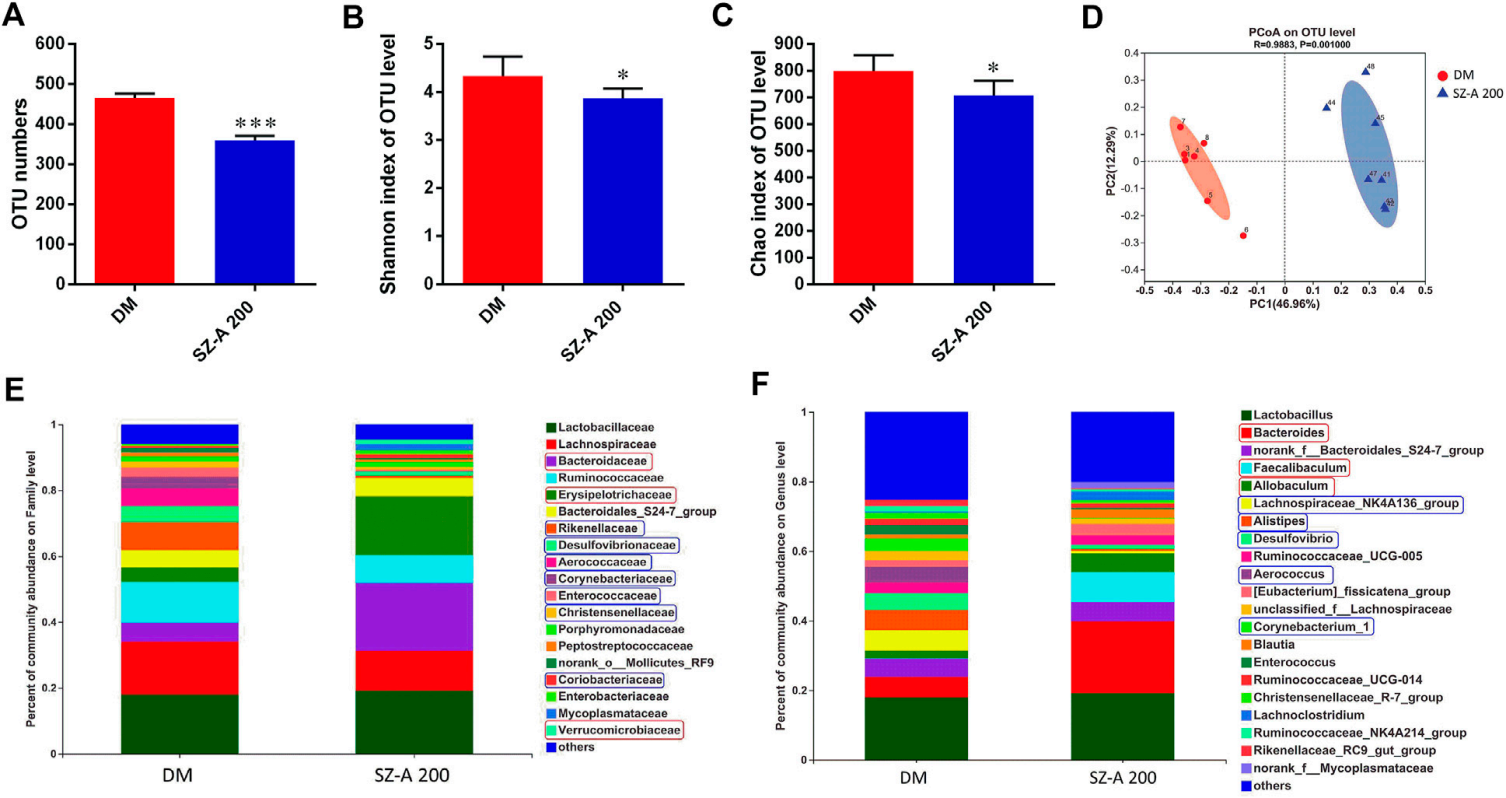
SZ-A modulates the composition of gut microbiota of KKAy mice. **(A)** Total OTU numbers. **(B)** Shannon index. **(C)** Chao index. **(D)** PCoA. **(E)** Percentage of community abundance of microbiota at the family level. **(F)** Percentage of community abundance of microbiota at the genus level (upregulated, red box; downregulated, Blue box) data are shown as mean ± SEM, *n* = 7. *p < 0.05, ***p < 0.001 *vs*. DM. DM, diabetic model group, SZ-A 100, SZ-A low-dose-treated group, SZ-A 200, SZ-A-high-dose-treated group.

The effects of high-dose SZ-A (SZ-A 200, 200 mg/kg) on intestinal microbiota composition were examined by Illumina sequencing-based analysis of bacterial 16S ribosomal RNA in fecal samples collected at the end of 8 weeks treatment. Compared to the DM group, the operational taxonomic unit (OTU) numbers were reduced in the SZ-A 200 group (Figure 2A; *p* < 0.001). The Shannon and Chao indices reflect the diversity and richness of gut microbiota, respectively. As shown in Figures 2B,C, SZ-A diminished the indices of Shannon and Chao (*p* < 0.05, *p* < 0.05). Unweighted Unifrac principal coordinate analysis (PCoA) based on OTU levels revealed distinct clustering of microbiota composition in each group (Figure 2D). Multivariate analysis of variance of PCoA matrix scores revealed that the microbiota community of mice in the SZ-A 200 group differed from that of the DM group (*p* < 0.001). Additionally, the bacterial community of SZ-A 200-treated mice differed from that of the DM group. Taxonomic profiling at the family level revealed that SZ-A treatments elevated the abundance of *Bacteroidaceae*, *Erysipelotrichaceae*, and *Verrucomicrobia* and reduced that of *Rikenellaceae*, *Desulfovibrionaceae*, and *Aerococcaceae* compared with DM mice (Figure 2E). Similar results were also observed at the genus level. SZ-A 200 decreased the abundance of Alistipes, Desulfovibrio, and *Aerococcus*, and increased the abundance of *Bacteroides*, *Faecalibaculum*, and *Allobaculum* compared with the DM group (Figure 2F). Collectively, these findings indicate that SZ-A 200 modulates the composition of gut microbiota.

**FIGURE 3:**
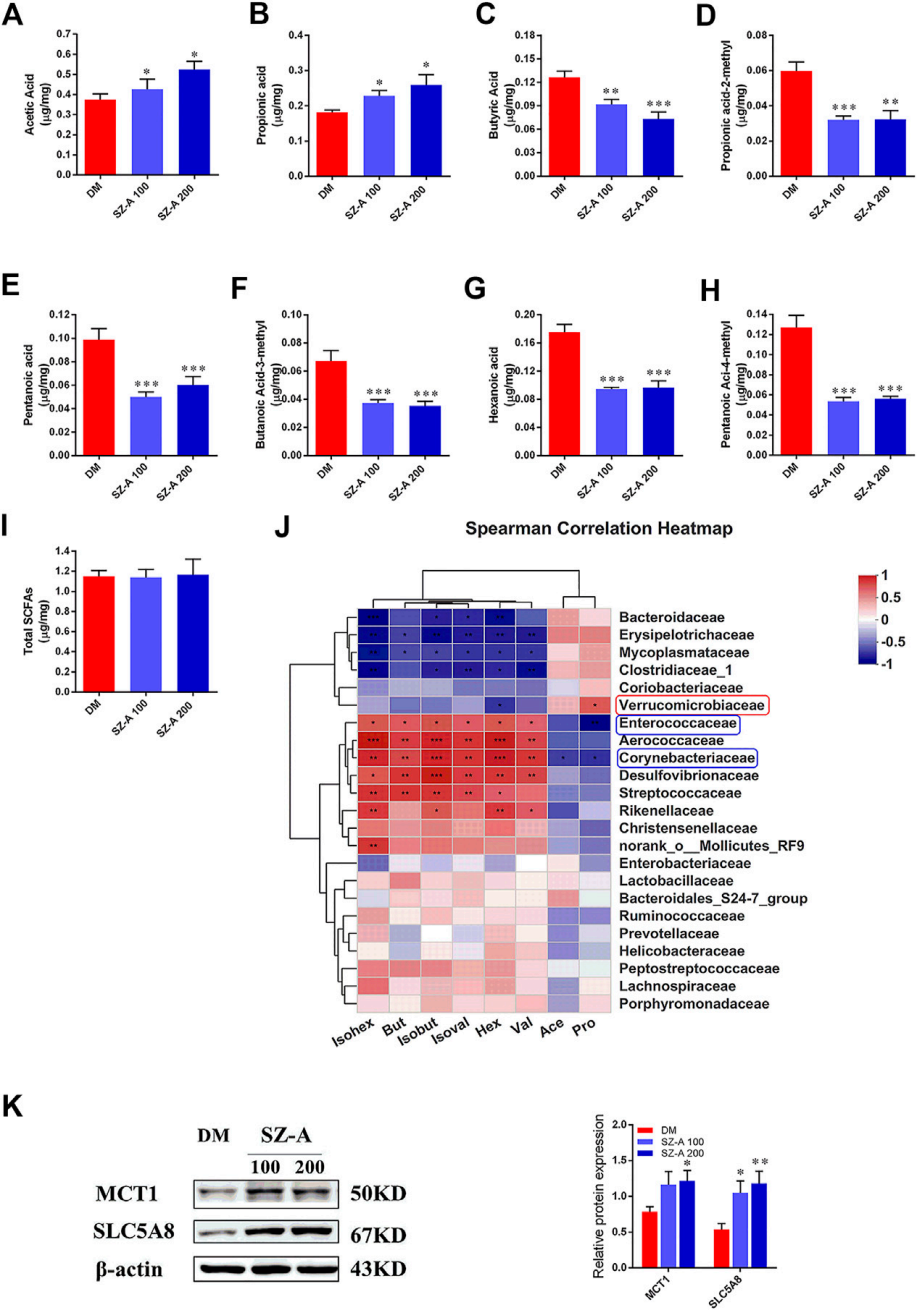
SZ-A alters SCFA composition of fecal samples in KKAy mice. Fecal SCFA concentration, including **(A)** acetic acid, **(B)** propionic acid, **(C)** butyric acid, **(D)** propionic acid-2-methyl, **(E)** pentanoic acid, **(F)** butanoic-acid-3-methyl, **(G)** hexanoic acid, **(H)** pentanoic-acid-4-methyl and **(I)** total SCFAs. **(J)** Correlation analysis of SCFAs and specific microbiota at the family level. The *p* values are shown in different colors in the diagram. The blue represents negative correlation, and red represents positive correlation. *n* = 7–8. **(K)** MCT1 and SLC5A8 protein abundance in the ileum tissue. The blots shown are representative images. *n* = 6. Data are mean ± SEM, *p < 0.05, **p < 0.01, ***p < 0.001 *vs*. DM. DM, diabetic model group, SZ-A 100, SZ-A low-dose-treated group, SZ-A 200, SZ-A-high-dose-treated group.

Short-chain fatty acids (SCFAs) are generated in the gut by bacterial fermentation of dietary fiber. Fecal SCFA concentrations were quantified to assess the impact of SZ-A on bacterial metabolic activity in KKAy mice. The concentrations of acetic (*p* < 0.05, *p* < 0.05) and propionic acids (*p* < 0.05, *p* < 0.05) were elevated in both SZ-A-treated groups (Figures 3A,B), whereas butyric, isobutyric (propionic acid-2-methyl), pentanoic, isopentanoic (butanoic acid-3-methyl), hexanoic, and isohexanoic (pentanoic acid-4-methyl) acids decreased in the SZ-A-treated groups compared to the DM group (Figures 3C–H). No significant changes were observed in total SCFA concentration between the SZ-A-treated and DM groups (Figure 3I). Subsequently, the relationship between fecal SCFAs and intestinal bacterial at the family level was analyzed (Figure 3J). The results showed that *Enterococcaceae* and *Corynebacteriaceae*, *Aerococcaceae*, *Desulfovibrionaceae*, and *Rikenellaceae* were positively correlated with the decreased SCFAs, including butyric, isobutyric, hexanoic, isohexanoic, pentanoic, and isopentanoic acids (Figure 3J). *Enterococcaceae* and *Corynebacteriaceae* abundance was negatively correlated with propionic acid, *Corynebacteriaceae* showed no correlation with acetic acid, and *Verrucomicrobiaceae* was positively correlated and propionic acid was observed (Figure 3J). Several transport systems play a role in the cellular uptake of SCFAs in the gut, including monocarboxylate transporter-1 (MCT1) and sodium-coupled monocarboxylate transporter 1 (SMCT1) (SLC5A8). As the transporters responsible for the entry and transcellular transfer of these bacterial products in epithelium are critical determinants of gut function, we detected MCT1 and SLC5A8 protein expression levels in the ileum tissue after SZ-A treatment in KKAy mice. The results showed that MCT1 and SLC5A8 protein levels in ileum from SZ-A-treated mice were significantly increased compared with the DM group.

### SZ-A Alleviates Ileal Inflammatory Injury and Pro-Inflammatory Macrophage Infiltration in KKAy Mice

**FIGURE 4:**
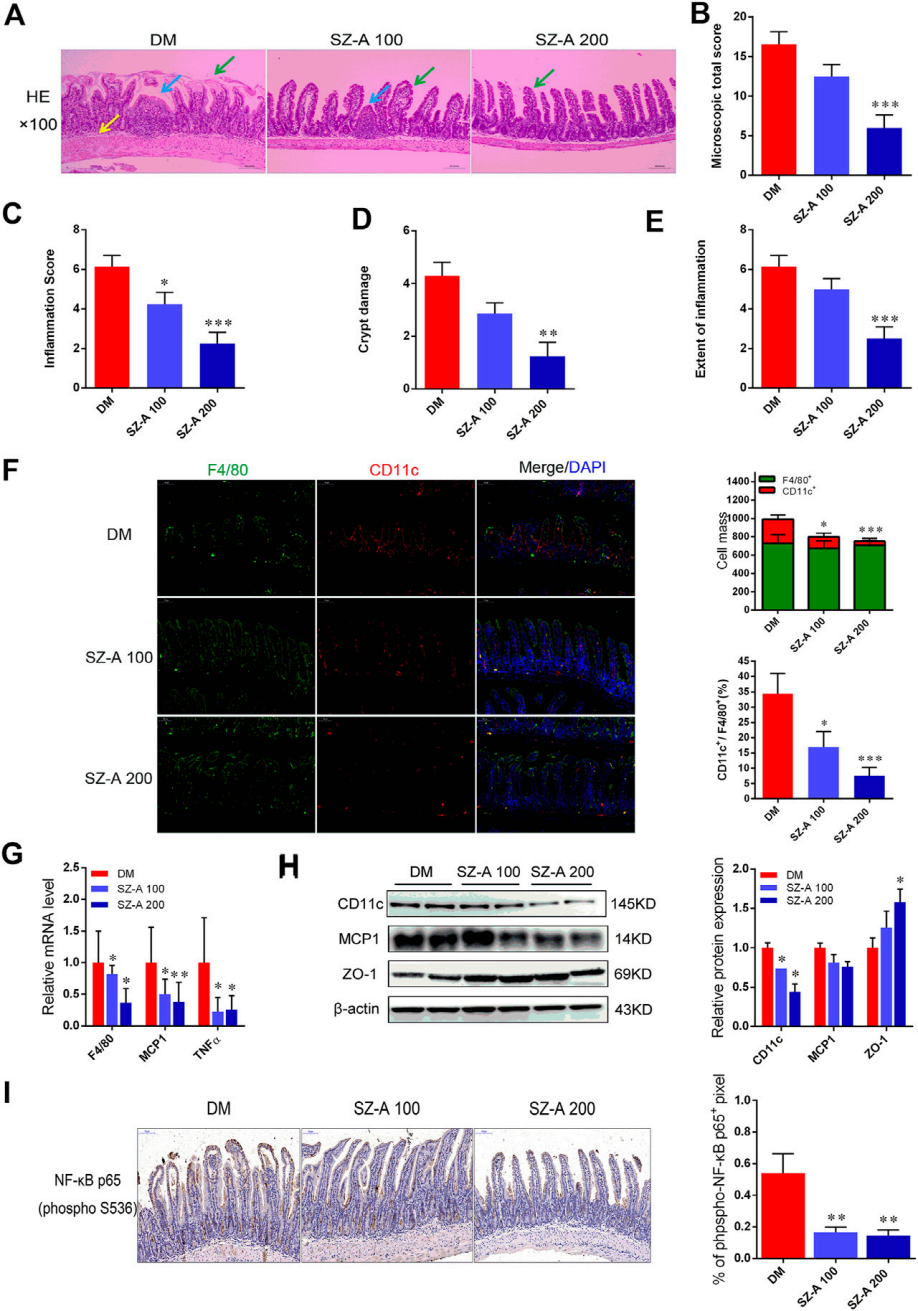
SZ-A alleviates ileal inflammatory injury and pro-inflammatory macrophage infiltration in KKAy mice. **(A)** Representative H&E staining of the ileal section (scale bar, 200 µm). **(B)** Histopathological assessment of total score. **(C)** Inflammation score. **(D)** Crypt damages score. **(E)** Extent of inflammation score of ileal sections. **(F)** Representative F4/80 (green), CD11c (red) and DAPI (blue) immunofluorescence staining of ileum tissues (Scale bar, 100 µm). The histograms indicate quantification of F4/80 (F4/80^+^) or CD11c (CD11c^+^) positive cells per field and percentages of CD11c positive cells in macrophages (F4/80^+^) (n = 5 per group). **(G)** Real-time PCR analysis of F4/80, MCP1 and TNF-α levels in ileal homogenates (*n* = 5 per group). **(H)** CD11c, MCP1 and ZO-1 protein abundant levels. The blots shown were representative images. **(I)** Sections of the ileum were stained with anti-NF-κB p65 (p-S536), the histograms indicate quantification of p-NF-κB p65 positive area per field (*n* = 5 per group). Data were mean ± SEM, *p < 0.05, **p < 0.01, ***p < 0.001 *vs*. DM. DM, diabetic model group, SZ-A 100, SZ-A low-dose-treated group, SZ-A 200, SZ-A-high-dose-treated group.

Considering microbial SCFAs production (especially acetate, propionate, and butyrate) is essential for gut integrity by regulating the mucus production, providing fuel for epithelial cells and effects on mucosal immune function, the histological alteration of ileum tissue were evaluated by hematoxylin and eosin (H&E) staining. As shown in Figure 4A, in the DM group, a dense inflammatory cellular infiltrate was present in the mucosa and submucosa and crypts showed typical shortening. Focal crypts were lost and the surface epithelium was damaged (Figure 4A). Microscopic total score and scores for the three features (inflammation, extent of inflammation, and crypt damage) were given for each group (Figures 4B–E). Inflammation scores were significantly reduced by both doses of SZ-A (*p* < 0.05, *p* < 0.001). Moreover, microscopic total score, crypt damage score, and inflammation score were significantly reduced in the SZ-A 200 group (*p* < 0.001, *p* < 0.01, and *p* < 0.001). Collectively, long-term SZ-A treatment prevented the development of inflammation and restored ileal barrier integrity in diabetic KKAy mice.

The KKAy mice were fed a high-fat diet (HFD) to induce diabetic syndrome. Given the critical role of M1 macrophages in HFD-induced intestinal inflammation, in parallel to those histological changes, macrophage-specific F4/80 and CD11c expression was measured to verify whether SZ-A treatment was able to modulate macrophage infiltration in the ileum tissue. As shown in Figure 4F, compared to the DM group, fewer pro-inflammatory CD11c-positive macrophages were observed in both doses of SZ-A-treated groups (*p* < 0.05, *p* < 0.001). This indicates a reduced inflammatory state after SZ-A treatment, and fully consistent with this result, we found downregulated mRNA expression of F4/80 (*p* < 0.05, *p* < 0.05) and multiple pro-inflammatory factors, including MCP1 (*p* < 0.05, *p* < 0.01) and TNF-α (*p* < 0.05, *p* < 0.05; Figure 4G), and also reduced CD11c protein expression in the ileum of SZ-A-treated groups (*p* < 0.05, *p* < 0.05; Figure 4H), compared to the DM group. Given that inflammation damages gut permeability and integrity, we also detected the protein expression levels of zonula occludens-1 (ZO-1), an intestinal tight junction component. SZ-A 200 markedly elevated ZO-1 protein levels (*p* < 0.05) compared to the DM group.

Nuclear factor kappa B (NF-κB) is critically associated with the progression of inflammation and cell proliferation in the intestinal mucosa. Therefore, the effects of SZ-A on NF-κB activity on the ileal mucosa were investigated. The indices of the phosphorylated (p-NF-κB) p65-positive area were markedly reduced with SZ-A treatment (*p* < 0.01, *p* < 0.01; Figure 4I). These findings indicate that SZ-A significantly alleviated ileal inflammatory injury and attenuated the inflammatory state induced by pro-inflammatory macrophage infiltration of the ileum tissue in diabetic KKAy mice.

### SZ-A Attenuates Endotoxin Content, Pro-Inflammatory Cytokine, and Chemokine Levels in Serum of Diabetic KKAy Mice

Gut dysbiosis not only leads to increased intestinal permeability, but it also results in the translocation of bacterial products into circulation, inducing a state of chronic low-grade inflammation, such as LPS in HFD-induced diabetes. Considering the effects of SZ-A on modulating gut microbiota profiling and alleviating ileal inflammatory injury, serum endotoxin content, and levels of cytokines and chemokines were determined after SZ-A treatment of diabetic KKAy mice.

**FIGURE 5:**
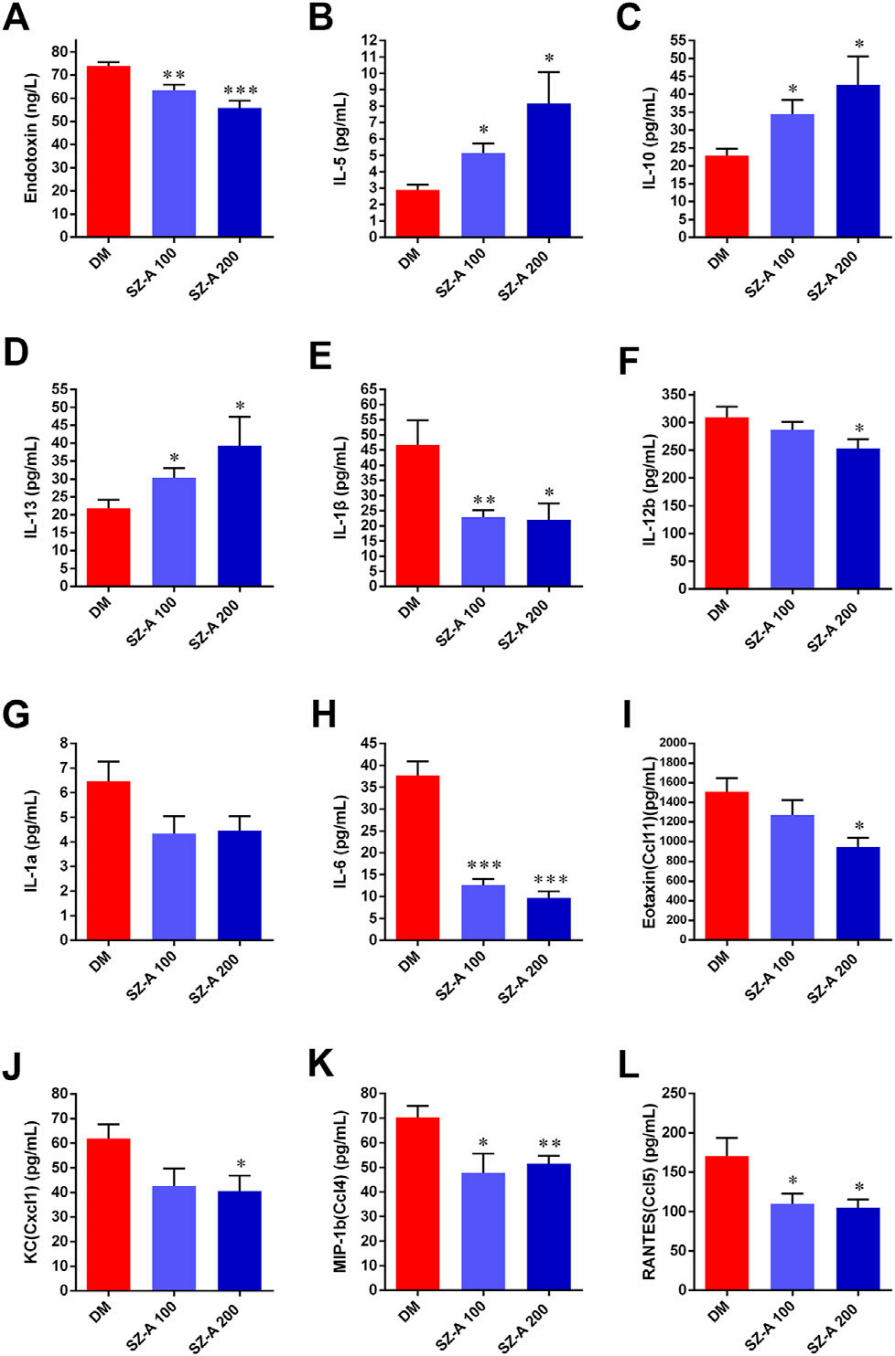
SZ-A improves inflammatory status in KKAy mice. **(A)** IL-5, **(B)** IL-10 **(C)** IL-13, **(D)** IL-1β, **(E)** IL-1a, **(F)** IL-6, **(G)** IL-12b, **(H)** Eotaxin (Ccl11), **(I)** KC(Cxcl1), **(J)** MIP-1b (Ccl4), **(K)** RANTES (Ccl5) and **(L)** Endotoxin. Data are expressed as the mean ± SEM, n = 8. *p < 0.05, **p < 0.01, ***p < 0.001 *vs*. DM. DM, diabetic model group, SZ-A 100, SZ-A low-dose-treated group, SZ-A 200, SZ-A-high-dose-treated group.

Compared with the DM group, both doses of SZ-A markedly diminished serum levels of the endotoxin content (*p* < 0.01, *p* < 0.001; Figure 5A), inflammatory cytokines and also chemokines, such as interleukin 1β (IL-1β, *p* < 0.01, *p* < 0.05; Figure 5E), IL-6 (*p* < 0.001, *p* < 0.001; Figure 5H), chemokine ligand 4 (CCL4) (*p* < 0.05, *p* < 0.01; Figure 5K), and CCL5 (*p* < 0.05, *p* < 0.05; Figure 5L). Additionally, serum levels of IL-12β (*p* < 0.05; Figure 5F), CCL11 (*p* < 0.05; Figure 5I), and CXCl1 (*p* < 0.05; Figure 5J) were also significantly reduced in the SZ-A 200-treated group. However, serum levels of anti-inflammatory cytokines IL-10 (*p* < 0.05, *p* < 0.05; Figure 5C) and IL-13 (*p* < 0.05, *p* < 0.05; Figure 5D) serum were markedly elevated.

### Functional Enrichment Analysis of Differentially Abundant Proteins in the Ileum after SZ-A Treatment

**FIGURE 6:**
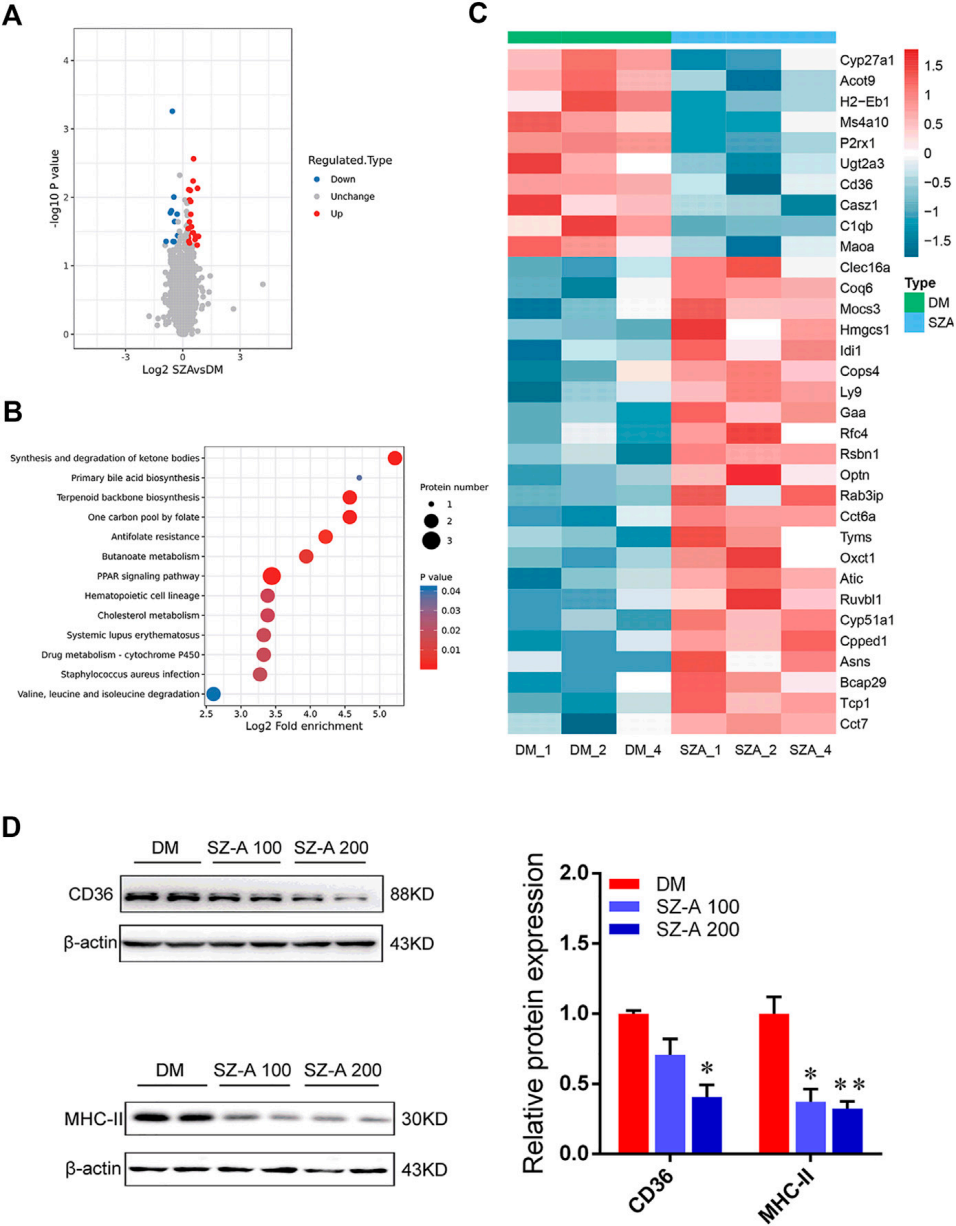
Functional enrichment analysis of differentially abundant proteins in the ileum tissues of KKAy mice after long-term SZ-A treatment. **(A)** Volcano plot shows the differentially abundant proteins in the ileum of SZ-A-treated group (FDR <0.01). Significantly differentially abundant proteins were color-coded: red indicates upregulated proteins, blue shows downregulated proteins. Two clusters consisting of 24 upregulated and 10 downregulated proteins. **(B)** Bubble diagram of enrichment of differentially abundant proteins in KEGG. The results of the first 20 classifications with the most significant enrichment were given in the bubble diagram (*p* < 0.05). **(C)** The heat map from three independent proteomic analyses of testes from the DM and SZA group. **(D)** Validated the downregulated proteins in ileum of DM and SZ-A-treated groups by Western blotting. CD36 and MHC-II protein abundant levels. The blots shown were representative images. Data are mean ± SEM, *n* = 6. *p < 0.05, **p < 0.01 *vs*. DM. DM, diabetic model group, SZA, SZ-A-high-dose-treated group.

Proteomics were used to determine the molecular characteristics of the ileum in the high-dose SZ-A-treated group (SZA) and DM group in KKAy mice. Refer to the supplementary data Supplementary Figure S1 , liquid chromatography tandem mass spectrometry identified 208,219 secondary spectra. A total of 42,677 matching effective spectra were obtained. Using a false discovery rate (FDR) < 1% at the peptide and protein levels, 25,060 of the 25,816 peptides were identified as specific, and 4352 of the 5043 proteins were quantifiable (Supplementary Figure S1A). A total of 34 proteins were differentially expressed (fold change >1.2, *p* < 0.05; Figure 6A) between the DM group and SZA group, of which 24 proteins were upregulated and 10 were downregulated. To determine the characteristics of the differentially expressed proteins, we annotated the subcellular localization, Clusters of Orthologous Group, and Gene Ontology (GO) of the 34 proteins. Annotation of the subcellular localization showed that 44.12% of all identified differentially expressed proteins were localized in the cytoplasm, 14.71% in the plasma membrane, 14.71% in the mitochondria, 11.76% in the nucleus, 8.82% in the extracellular space, and 5.88% in the endoplasmic reticulum (Supplementary Figure S1B). Most differentially abundant proteins participated in and regulated the cellular and metabolic processes (Supplementary Figure S1C). COG functional classification revealed that most of these differentially abundant proteins played a role in posttranslational modification, protein turnover, and chaperones (Supplementary Figure S1D).

Bioinformatics analysis was performed to identify the main biological pathways and functional categories of the differentially abundant proteins (fold change >1.2; *p* < 0.05). Kyoto Encyclopedia of Genes and Genomes (KEGG) analysis showed that the most significantly altered pathways were involved in primary bile acid biosynthesis, peroxisome proliferator-activated receptor (PPAR) signaling pathway, synthesis and degradation of ketone bodies, and terpenoid backbone biosynthesis (Figure 6B). We identified 34 abundant proteins that were mainly involved in the above-mentioned pathways (Figure 6C). These proteins included downregulation of cluster of differentiation 36 (CD36), a protein related to the PPAR pathway; CYP27a1, the representative differentially abundant protein related to primary bile acid biosynthesis; and histocompatibility 2 class II antigen E beta, which is critical in the antigen processing and presentation pathway and is also described as major histocompatibility complex class II (MHC II). The expression level of these key regulators identified *via* proteomics was also confirmed by Western blotting. The results showed the level of CD36 (SZ-A 200 group; *p* < 0.05) and MHC II (*p* < 0.05, *p* < 0.01) were significantly reduced in SZ-A-treated groups compared to the DM group (Figure 6D), which is consistent with proteomics analysis.

## Discussion

In this study, we evaluated the therapeutic efficacy of SZ-A in glucose and lipid metabolism *in vivo* in the diabetic KKAy mice. We found that SZ-A ameliorated glucose metabolism and enhanced the insulin response to oral glucose tolerance tests and elevated active GLP-1 levels in diabetic KKAy mice. We suspect that the increased glucose-stimulated insulin secretion might be primarily linked to the improved β-cell function after SZ-A treatment, or related to the elevated blood levels of active GLP-1 in KKAy mice. However, the fact that oral (current study) but not intraperitoneal injection of glucose was associated with improved glucose tolerance in KKAy mice after SZ-A treatment indicated the potential involvement of incretin hormones.

GLP-1, an incretin hormone released in response to the ingestion of nutrients, acts as a hypoglycemic hormone to improve postprandial glucose homeostasis by enhancing meal-induced insulin secretion. GLP-1 activity is mediated by the GLP-1 receptor (GLP-1R). GLP-1Rs are highly expressed in pancreatic β-cells and other tissues including neurons in specific central brain regions, the kidney, and the gut tract (Gray et al., 2020). Findings from the clinical trials have also revealed that the administration of GLP-1R agonists (GLP-1RAs) induces weight loss in addition to glucose improvement (Salehi and Purnell, 2019). In our study, we also found that both doses of SZ-A induced significant weight loss at the end of long-term treatment, whereas the glycemic-lowering effects of GLP-1RAs were mainly attributed to endocrine actions at the level of pancreatic islets. Thus, we evaluated changes in α- and β-cell mass or distribution to analyze the functional state of islets after SZ-A treatment. In Supplementary Figure S2, immunofluorescence staining showed that the glucagon-positive area was significantly scattered around the islets in the DM group, suggesting the imbalanced distribution of α-cells. SZ-A decreased the ratio of glucagon-positive area and improved the distribution of α-cells.

GLP-1 is predominantly secreted by neuroendocrine L-cells in the gut. L cells, which produce both GLP-1 and peptide YY, are predominantly expressed in the distal ileum. In humans, prebiotic treatment increases microbiome diversity, which in turn may modulate levels of GLP-1 (Grasset et al., 2017). Moreover, with ileal transposition surgery, Lcells located in the transposed ileum are rapidly stimulated by ingested nutrients to produce GLP-1 (Oh et al., 2016). Given the close anatomical proximity, gut microbiota could potentially alter the nutrient-sensing capacity of enteroendocrine cells and subsequent gut peptide release. Additionally, the major components of SZ-A (DNJ, FAG, and DAB) were mainly distributed in the gastrointestinal tract. Therefore, we hypothesized that SZ-A caused the altered active GLP-1 levels, possibly by regulating gut microbiota composition. No study has investigated the effect of SZ-A on the distal ileum microbiota, a site that is crucial for intestinal sensing and absorption of nutrients. Therefore, in this study, we evaluated the interaction between SZ-A and ileal microbiota. Then the effects of SZ-A on the gut microbiota were investigated.

We first characterized the microbiota composition from DM and the high dose-SZ-A treated group (SZ-A 200). Mice were sacrificed and the luminal contents were collected from the ileum (nearly 12 cm distal to the pyloric sphincter). SZ-A increased the abundance of *Bacteroidaceae*, *Erysipelotrichaceae*, and *Verrucomicrobia*. SZ-A also markedly decreased the abundance of *Rikenellaceae*, *Desulfovibrionaceae*, and *Aerococcaceae* at the family level compared with DM mice (Figure 2E). Similar results were observed at the genus level. SZ-A reduced the abundance of *Alistipes*, *Desulfovibrio*, and *Aerococcus*, and increased the abundance of *Bacteroides*, *Faecalibaculum*, and *Allobaculum*. *Bacteroidetes* and *Verrucomicrobia* play crucial roles in producing SCFAs (Harris et al., 2012). We also detected measurable fecal SCFA concentrations in the DM and SZ-A-treated groups, which is consistent with previous studies characterizing the ileal microbiota composition of diabetic KKAy mice. We observed elevated propionate and acetate in the SZ-A-treated groups (Figures 3A,B). Propionate and acetate are two of the main SCFAs that are produced by bacteria as a result of resistant starch fermentation. SZ-A-induced microbiota changes facilitated propionate and acetate production in the ileum, which might explain the elevated basal and glucose-stimulated blood active GLP-1 levels in diabetic KKAy mice after SZ-A treatment.

The microbiota-derived metabolites in the luminal contents also represent a potential link to expression changes of nutrient sensors, ligand-receptors, or transporters. Figure 3 shows that with the exception of propionate and acetate, other SCFA concentrations in ileal luminal contents were decreased after SZ-A treatment. Several transport proteins are involved in the uptake of SCFAs in the gut, including MCT1 and SMCT1 (SLC5A8). These transporters are critical determinants of the entry and transcellular transfer of SCFAs into intestinal epithelium under physiological conditions and in disease states (Bauer et al., 2018). We detected the protein expression levels of these two transporters. There was significant upregulation in the expression of MCT1 and SMCT1 (SLC5A8) in the ileum of SZ-A-treated groups (Figure 3K), which is consistent with the decreased level of SCFAs following SZ-A treatment. Collectively, we determined a connection between SZ-A action and changes in ileal microbiota to regulate SCFA production, which in turn, affect glucose homeostasis through the regulation of GLP-1 secretion.

LPS is a major component of the cell wall of gram-negative bacteria and is considered an endotoxin when present in the blood. Increased LPS levels can induce a large number of proinflammatory responses and inflammatory cytokine release (Fuke et al., 2019). Of note, among the changes in the gut microbiota of KKAy mice after SZ-A treatment, the decreased abundance of LPS-containing *Desulfovibrionaceae* was observed. *Desulfovbrionaceae* is an endotoxin producer and has been linked to gut permeability (Clemente-Postigo et al., 2012). There is increasing evidence of the role of gut microbiota in various inflammatory diseases, especially those affecting gastrointestinal tract inflammation. Additionally, butyrate, propionate and acetate are SCFAs that are produced by bacteria as a result of resistant starch fermentation, and have anti-inflammatory properties (Blaak et al., 2020). Due to these effects of SZ-A on the composition of the gut microbiota, it is speculated that SZ-A treatment might repair the inflammatory damage of intestine by modulating the abundance of LPS and SCFA-producing gut microbiota.

Subsequently, H&E staining was performed on the ileum of KKAy mice after SZ-A treatment. Three different sections were studied for each animal. Histological inflammation was scored by two blinded investigators using a modified scoring system (Dieleman et al., 1998). Considering the degree of inflammation, the transmural vertical extent of inflammation, and the crypt damage score, related to the percentage of involvement of mucosal surface in each slide. As shown in Figures 4A–E, this finding supported the occurrence of damage of the ileal mucosal barrier in diabetic KKAy mice (DM group) and repaired ileal inflammatory damage after treatment with both doses of SZ-A. LPS production is also responsible for inducing monocyte- and macrophage-mediated inflammation in the intestine (Wang et al., 2020). Gut macrophages, which reside in the connective tissue underlying the gut epithelium, the lamina propria, are considered key players for the maintenance of intestinal homeostasis and inflammation (Yang et al., 2020). We observed that in ileal tissues, the percentage of macrophages (CD11c^+^/F4/80^+^) and the cell mass of CD11c-positive monocytes were significantly decreased with both doses of SZ-A compared with the control (Figure 4F). The degree of expression of F4/80, MCP-1, and TNFα mRNA in both SZ-A-treated groups was significantly decreased compared to the DM group. Similarly, the protein levels of CD11c and MCP1 in the ileum were also reduced by SZ-A treatment (Figures 4G,H). The NF-κB signaling pathway was also detected in the ileum. The phosphorylation of p65 and its nuclear translocation were significantly decreased by SZ-A treatment. These results suggest that SZ-A treatment ameliorate ileal inflammatory damage in KKAy mice with down-regulated inflammatory signaling pathways partly *via* reducing the monocyte recruitment in the iluem.

Over time, major inflammatory signals (e.g., NF-κB-dependent) become activated in diabetic KKAy mice, thereby stimulating pro-inflammatory cytokine secretion in the small intestine. This inflammatory state might subsequently exacerbate the disruption of the mucus layer barrier and increase epithelial permeability of the small intestine. We observed increased protein levels of the tight junction protein ZO-1 in the SZ-A-treated group, which can reflect the intestinal mucosal barrier function. Our results also confirmed the results that the intestinal mucosal barrier function was improved after treatment of KKAy mice with SZ-A. The persistent inflammatory state not only increases intestinal permeability but also the destruction of tight junction proteins attached to epithelial cells, increasing portal vein and systemic plasma LPS concentrations, and eventually promoting the development of systemic inflammation (Clemente-Postigo et al., 2012). Therefore, we subsequently determined the concentrations of endotoxin and inflammatory cytokines in the serum of KKAy mice. The serum endotoxin levels significantly decreased after both doses of SZ-A treatment, compared to the DM group. The serum levels of inflammatory factors (e.g., IL-1β, IL-6, CCL4, and CCL5) indicated that low-grade inflammation in diabetic KKAy mice decreased after SZ-A treatment, which corresponded to the endotoxin level. Thus, ileal inflammatory damage in the diabetic KKAy mice was positively associated with low-grade inflammation, which was effectively alleviated by SZ-A treatment.

The production of pro-inflammatory cytokines is related to the LPS-induced activation and polarization of macrophage. We observed there was significantly suppressed polarization of macrophages in the ileum after SZ-A treatment in diabetic KKAy mice. The molecular characteristics of the ileal tissues in the high-dose SZ-A and DM groups in diabetic KKAy mice were investigated through proteomics. Proteomics analysis revealed that monocyte differentiation into intestinal macrophages involves phenotypic changes with MHCII expression was downregulated by SZ-A treatment, which may be responsible for the improvement of ileal inflammatory damage after SZ-A treatment. Beyond that, the representative differentially downregulated abundant proteins related to PPAR pathway and fat digestion and absorption, ileal CD36 expression level was significantly decreased in SZ-A-treated groups (Figure 6D), which might be related to improved blood triglyceride levels.

## Conclusion

Here, we reported that long-term treatment of SZ-A (8 weeks) is sufficient to alter the microbiota composition in the ileum of diabetic KKAy mice. Our data, together with enriched literature, provide novel mechanistic insights into the role of SZ-A in mediating gut microbial community in ileal inflammatory damage and glucose metabolism.

## Data Availability

The datasets presented in this study can be found in online repositories. The names of the repository/repositories and accession number(s) can be found below: NCBI SRA; BioProject ID PRJNA701784. The mass spectrometry proteomics data have been deposited to the ProteomeXchange Consortium via the PRIDE [1] partner repository with the dataset identifier PXD024241.

## Abbreviations

Ccl11, Chemokine C-C-Motif Ligand 1; Ccl4, Chemokine C-C-Motif Ligand 4; Ccl5, Chemokine C-C-Motif Ligand 5; CXCl1, CXC chemokine ligand 1; CD36, cluster of differentiation 36; 1-DNJ, 1-deoxyno-jirimycin; DAB, 1,4-dideoxy-1, 4-iminod-D-arabinitol; FAG, fagomine; F4/80, adhesion G protein-coupled receptor E1; GLP-1, glucagon-like peptide 1; HbA1c, glycated hemoglobin; IL-1β, Interleukin 1β; IL-5, Interleukin 5; IL-10, Interleukin 10; IL-13, Interleukin 13; IL-1a, Interleukin 1a; IL-6, Interleukin 6; IL-12b, Interleukin 12b; LPS, lipopolysaccharides; MCT1, monocarboxylate transporter‐1; MHC II, major histocompatibility complex class II; MCP1, monocyte chemotactic protein 1; NF-κB, Nuclear factor kappa B; SCFAs, short-chain fatty acids; SLC5A8, sodium-coupled monocarboxylate transporter 1; T2DM, type 2 diabetes mellitus; TCM, Traditiona l Chinese Medicine; TNFα, tumor necrosis factor α; ZO-1, Zonula Occludens-1.

## References

[B1] Bauer P. V. Duca F. A. Waise T. M. Z. Rasmussen B. A. Abraham M. A. Dranse H. J. (2018). Metformin alters upper small intestinal microbiota that impact a glucose-SGLT1-sensing glucoregulatory pathway. Cel Metab. 27 (1), 101–117. 10.1016/j.cmet.2017.09.019 29056513

[B2] Blaak E. E. Canfora E. E. Theis S. Frost G. Groen A. K. Mithieux G. (2020). Short chain fatty acids in human gut and metabolic health. Beneficial Micro. 11 (5), 411–455. 10.3920/bm2020.0057 32865024

[B3] Cao H. Li C. Lei L. Wang X. Liu S. Liu Q. (2020). Stachyose improves the effects of berberine on glucose metabolism by regulating intestinal microbiota and short-chain fatty acids in spontaneous type 2 diabetic KKAy mice. Front. Pharmacol. 11, 578943. 10.3389/fphar.2020.578943 33192521 PMC7642818

[B4] Clemente-Postigo M. Queipo-Ortuño M. I. Murri M. Boto-Ordoñez M. Perez-Martinez P. Andres-Lacueva C. (2012). Endotoxin increase after fat overload is related to postprandial hypertriglyceridemia in morbidly obese patients. J. Lipid Res. 53 (5), 973–978. 10.1194/jlr.P020909 22394503 PMC3329396

[B5] Dieleman L. A. Palmen M. J. Akol H. Bloemena E. Peña A. S. Meuwissen S. G. (1998). Chronic experimental colitis induced by dextran sulphate sodium (DSS) is characterized by Th_1_ and Th_2_ cytokines. Clin. Exp. Immunol. 114 (3), 385–391. 10.1046/j.1365-2249.1998.00728.x 9844047 PMC1905133

[B6] Fuke N. Nagata N. Suganuma H. Ota T. (2019). Regulation of gut microbiota and metabolic endotoxemia with dietary factors. Nutrients 11 (10), 2277. 10.3390/nu11102277 PMC6835897 31547555

[B7] Ge X. Pan J. Liu Y. Wang H. Zhou W. Wang X. (2018). Intestinal crosstalk between microbiota and serotonin and its impact on gut motility. Curr Pharm Biotechnol. 19 (3), 190–195. 10.2174/1389201019666180528094202 29804531

[B8] Grasset E. Puel A. Charpentier J. Collet X. Christensen J. E. Tercé F. (2017). A specific gut microbiota dysbiosis of type 2 diabetic mice induces GLP-1 resistance through an enteric NO-dependent and gut-brain *Axis* mechanism. Cel Metab. 25 (5), 1075–1090. 10.1016/j.cmet.2017.04.013 28467926

[B9] Gray S. M. Xin Y. Ross E. C. Chazotte B. M. Capozzi M. E. El K. (2020). Discordance between GLP-1R gene and protein expression in mouse pancreatic islet cells. J. Biol. Chem. 295 (33), 11529–11541. 10.1074/jbc.RA120.014368 32554468 PMC7450118

[B10] Hamada Y. Goto M. Nishimura G. Nagasaki H. Seino Y. Kamiya H. (2020). The alpha-glucosidase inhibitor miglitol increases hepatic CYP7A1 activity in association with altered short-chain fatty acid production in the gut of obese diabetic mice. Metab. Open 5, 100024. 10.1016/j.metop.2020.100024 PMC7424806 32812937

[B11] Harris K. Kassis A. Major G. Chou C. J. (2012). Is the gut microbiota a new factor contributing to obesity and its metabolic disorders? J. Obes. 2012, 1. 10.1155/2012/879151 PMC3270440 22315672

[B12] Kahn S. E. Cooper M. E. Del Prato S. (2014). Pathophysiology and treatment of type 2 diabetes: perspectives on the past, present, and future. Lancet 383 (9922), 1068–1083. 10.1016/S0140-6736(13)62154-6 24315620 PMC4226760

[B13] Kuti D. Winkler Z. Horváth K. Juhász B. Paholcsek M. Stágel A. (2020). Gastrointestinal (non-systemic) antibiotic rifaximin differentially affects chronic stress-induced changes in colon microbiome and gut permeability without effect on behavior. Brain Behav. Immun. 84, 218–228. 10.1016/j.bbi.2019.12.004 31821847

[B14] Li C. N. Wang X. Lei L. Liu M. Z. Li R. C. Sun S. J. (2020). Berberine combined with stachyose induces better glycometabolism than berberine alone through modulating gut microbiota and fecal metabolomics in diabetic mice. Phytotherapy Res. 34 (5), 1166–1174. 10.1002/ptr.6588 PMC7216932 31833107

[B15] Liu S. N. Liu Q. Sun S. J. Li C. Huan Y. Shen Z. F. (2019a). Anti-diabetic effects of the fraction of alkaloids from Ramulus Mori, an innovative Sangzhi alkaloids as an α-glucosidase inhibitor. Yao Xue Xue Bao 54 (7), 1233.

[B16] Liu Z. Yang Y. Dong W. Liu Q. Wang R. Pang J. (2019b). Investigation on the enzymatic profile of mulberry alkaloids by enzymatic study and molecular docking. Molecules 24 (9), 1776. 10.3390/molecules24091776 PMC6539310 31071910

[B17] Oh T. J. Ahn C. H. Cho Y. M. (2016). Contribution of the distal small intestine to metabolic improvement after bariatric/metabolic surgery: lessons from ileal transposition surgery. J. Diabetes Investig. 7 (Suppl. 1), 94–101. 10.1111/jdi.12444 PMC4854512 27186363

[B18] Salazar J. Angarita L. Morillo V. Navarro C. Martínez M. S. Chacín M. (2020). Microbiota and diabetes mellitus: role of lipid mediators. Nutrients 12 (10), 3039. 10.3390/nu12103039 PMC7600362 33023000

[B19] Salehi M. Purnell J. Q. (2019). The role of glucagon-like peptide-1 in energy homeostasis. Metab. Syndr. Relat. Disord. 17 (4), 183–191. 10.1089/met.2018.0088 30720393 PMC6610028

[B20] Shao J. Liu Y. Wang H. Luo Y. Chen L. (2020). An integrated fecal microbiome and metabolomics in T2DM rats reveal antidiabetes effects from host-microbial metabolic *Axis* of EtOAc extract from *Sophora* flavescens. Oxi. Med. Cell Longev. 2020, 1805418. 10.1155/2020/1805418 PMC7273480 32566075

[B21] Wang L. Gong Z. Zhang X. Zhu F. Liu Y. Jin C. (2020). Gut microbial bile acid metabolite skews macrophage polarization and contributes to high-fat diet-induced colonic inflammation. Gut Microbes 12 (1), 1819155–1819220. 10.1080/19490976.2020.1819155 PMC7553752 33006494

[B22] Wei Y. Liu B. Sun J. Lv Y. Luo Q. Liu F. (2015). Regulation of Th17/Treg function contributes to the attenuation of chronic airway inflammation by icariin in ovalbumin-induced murine asthma model. Immunobiology 220 (6), 789–797. 10.1016/j.imbio.2014.12.015 25613226

[B23] Xiao S. Liu C. Chen M. Zou J. Zhang Z. Cui X. (2020). Scutellariae radix and coptidis rhizoma ameliorate glycolipid metabolism of type 2 diabetic rats by modulating gut microbiota and its metabolites. Appl. Microbiol. Biotechnol. 104 (1), 303–317. 10.1007/s00253-019-10174-w 31758238

[B24] Yang S. Mi J. Liu Z. Wang B. Xia X. Wang R. (2017). Pharmacokinetics, tissue distribution, and elimination of three active alkaloids in rats after oral administration of the effective fraction of alkaloids from Ramulus Mori, an innovative hypoglycemic agent. Molecules 22 (10), 1616. 10.3390/molecules22101616 PMC6151740 28954438

[B25] Yang Y. Li L. Xu C. Wang Y. Wang Z. Chen M. (2020). Cross-talk between the gut microbiota and monocyte-like macrophages mediates an inflammatory response to promote colitis-associated tumourigenesis. Gut. 2020, 320777. 10.1136/gutjnl-2020-320777 PMC8292576 33122176

[B26] Zheng Y. Ding Q. Zhang L. Gou X. Wei Y. Li M. (2020). The effect of traditional Chinese medicine on gut microbiota in adults with type 2 diabetes. Medicine 99 (38), e22233. 10.1097/md.0000000000022233 32957365 PMC7505378

