# Supplementary material for: Ramulus Mori (Sangzhi) Alkaloids (SZ-A) Ameliorate Glucose Metabolism Accompanied by the Modulation of Gut Microbiota and Ileal Inflammatory Damage in Type 2 Diabetic KKAy Mice
Source: Front Pharmacol. 2021 Apr 15;12:642400. doi: 10.3389/fphar.2021.642400 (PMC8082153; doi:10.3389/fphar.2021.642400)
Supplement: Supplementary file 1 [file datasheet1.docx]

Supplementary Material

# Supplementary Methods

# 1.Quantitative real-time PCR

# Briefly, RNA was isolated from ileum tissues using TRizol reagent (15596018; Life Technology, USA) and reverse transcribed with TransScript® first-strand cDNA Synthesis SuperMix (AT311; TransGen Biotec, Beijing, China) based on the manufacturer’s protocols. Quantitative real-time PCR was conducted using TransStart® Tip Green qPCR SuperMix (AQ141; TransGen Biotec, Beijing, China) on 7900 Real-Time PCR System (Applied Biosystems, USA). Gene expression levels were normalized to those of β-actin.

# 2.Tandem Mass Tagging (TMT) proteomics analysis

# 2.1 Protein Extraction

# The mouse ileums were grinded by liquid nitrogen into cell powder and then transferred to a 5-mL centrifuge tube. After that, four volumes of lysis buffer (8 M urea, 1% Protease Inhibitor Cocktail) was added to the cell powder, followed by sonication three times on ice using a high intensity ultrasonic processor (Scientz). The remaining debris was removed by centrifugation at 12,000g at 4 °C for 10 min. Finally, the supernatant was collected and the protein concentration was determined with BCA kit according to the manufacturer’s instructions.

# 2.2 Trypsin Digestion

# For digestion, the protein solution was reduced with 5 mM dithiothreitol for 30 min at 56 °C and alkylated with 11 mM iodoacetamide for 15 min at room temperature in darkness. The protein sample was then diluted by adding 100 mM TEAB to urea concentration less than 2M. Finally, trypsin was added at 1:50 trypsin-to-protein mass ratio for the first digestion overnight and 1:100 trypsin-to-protein mass ratio for a second 4h digestion.

# 2.3 TMT Labeling

# After trypsin digestion, peptide was desalted by Strata X C18 SPE column (Phenomenex) and vacuum-dried. Peptide was reconstituted in 0.5 M TEAB and processed according to the manufacturer’s protocol for TMT kit. Briefly, one unit of TMT reagent were thawed and reconstituted in acetonitrile. The peptide mixtures were then incubated for 2h at room temperature and pooled, desalted and dried by vacuum centrifugation.

# 2.4 HPLC Fractionation

# The tryptic peptides were fractionated into fractions by high pH reverse-phase HPLC using Agilent 300Extend C18 column (5 μm particles, 4.6 mm ID, 250 mm length). Briefly, peptides were first separated with a gradient of 8% to 32% acetonitrile (pH 9.0) over 60 min into 60 fractions. Then, the peptides were combined into 18 fractions and dried by vacuum centrifuging.

# 2.5 LC-MS/MS Analysis

# The tryptic peptides were dissolved in 0.1% formic acid (solvent A), directly loaded onto a home-made reversed-phase analytical column (15-cm length, 75 μm i.d.). The gradient was comprised of an increase from 6% to 23% solvent B (0.1% formic acid in 98% acetonitrile) over 26 min, 23% to 35% in 8 min and climbing to 80% in 3 min then holding at 80% for the last 3 min, all at a constant flow rate of 400 nL/min on an EASY-nLC 1000 UPLC system. The peptides were subjected to NSI source followed by tandem mass spectrometry (MS/MS) in Q ExactiveTM Plus (Thermo) coupled online to the UPLC. The electrospray voltage applied was 2.0 kV. The m/z scan range was 350 to 1800 for full scan, and intact peptides were detected in the Orbitrap at a resolution of 70,000. Peptides were then selected for MS/MS using NCE setting as 28 and the fragments were detected in the Orbitrap at a resolution of 17,500. A data-dependent procedure that alternated between one MS scan followed by 20 MS/MS scans with 15.0s dynamic exclusion. Automatic gain control (AGC) was set at 5E4. Fixed first mass was set as 100 m/z.

# 2.6 Data Analysis

# The raw data were processed by GO Annotation (www. http://www.ebi.ac.uk/GOA/), Domain Annotation (InterProScan) and KEGG Pathway Annotation (KEGG online service tools KAAS mapper).

# 3.Immunofluorescent assay

# All mice were sacrificed through cervical dislocation and the pancreas was dissected to prepare 5-μm paraffin slides, which were stained against insulin (MAB1417; R&D Systems, USA) and glucagon (ab92517; Abcam, USA) (n = 5).

# Supplementary Figures and Tables

## Supplementary Figures

**
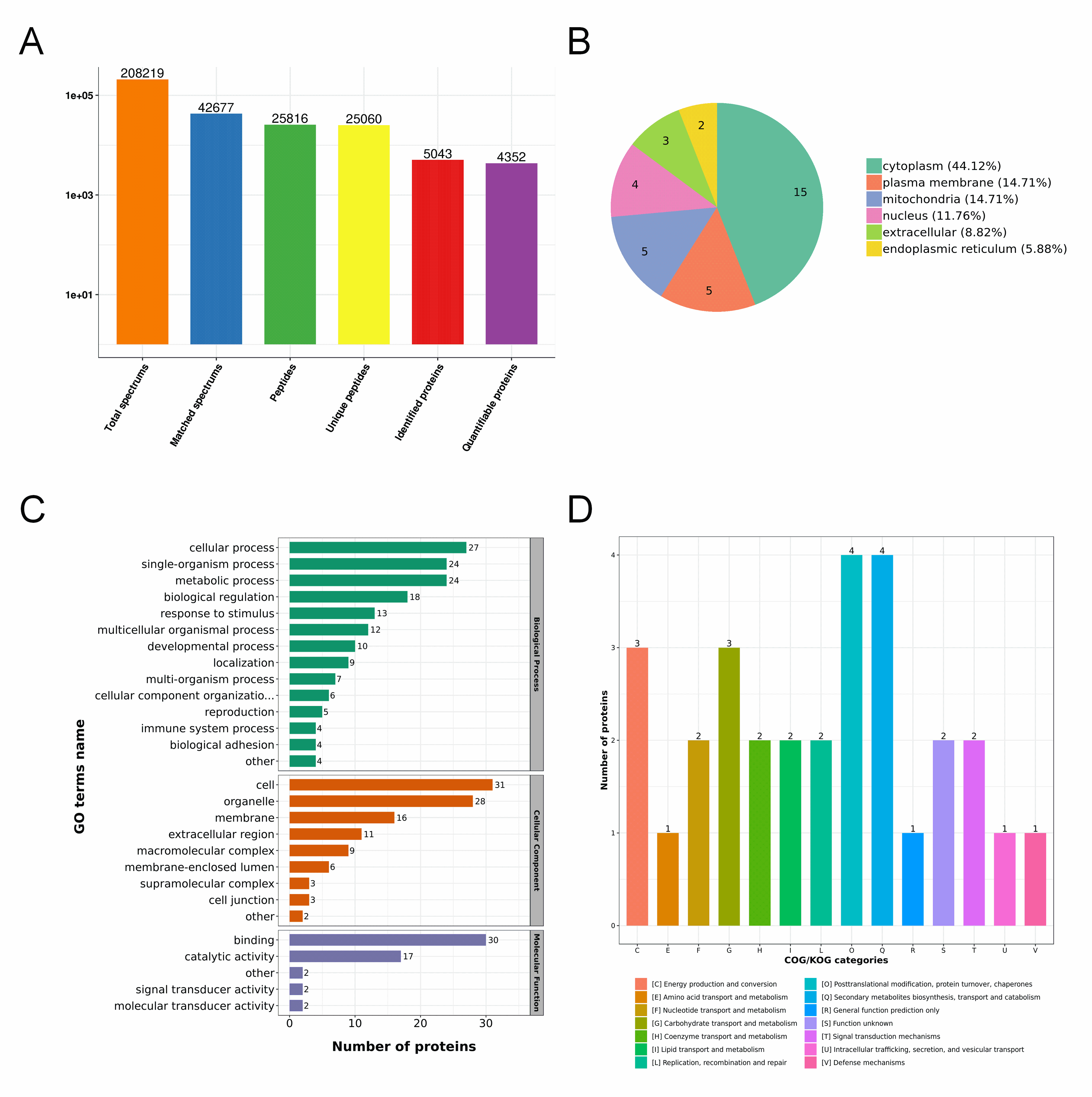
**

**Supplementary Figure 1. Analysis of differentially abundant proteins in ileum tissues.** (A) Protein spectrum analysis results of ileum. (B) Distribution map of Subcellular Localization of differentially abundant proteins. (C) Statistical distribution map of differentially abundant proteins in GO secondary classification. GO comments fall into three broad categories: Biological Processes (BP, green), Cell Composition (CC, orange), and Molecular Function (MF, purple). (D) Classification and distribution map of COG function of differentially abundant proteins.


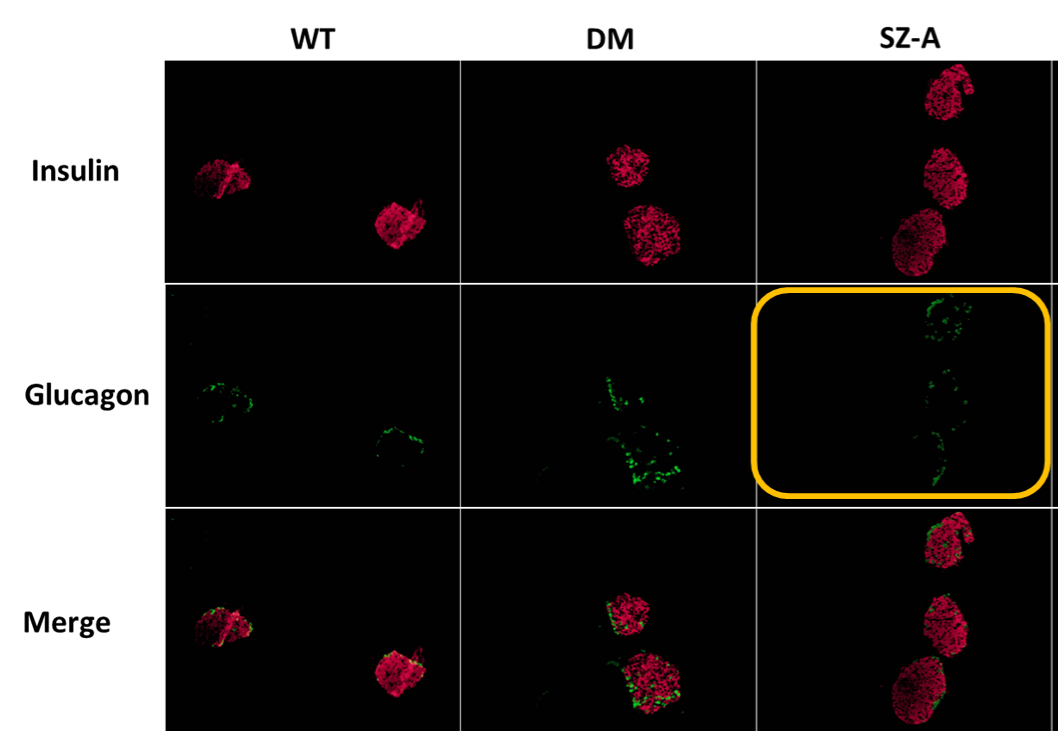


**Supplementary Figure 2. Representative images of insulin and glucagon immunofluorescence staining in pancreatic islets of the normal C57 mice and the diabetic KKAy mice after SZ-A treatment.** Insulin is shown in red, and glucagon in green. Magnification of all images is 400×. WT, age-matched normal C57 mice, DM, diabetic model group, SZ-A, SZ-A-high dose-treated group.

**Supplementary Table**

**Supplementary Table S1. The information for the PCR primers used in the present study.**

| **Gene** | **5’-Sense primer-3’** | **5’-Antisense primer-3’** | **Gene Bank** |
| --- | --- | --- | --- |
|  |  |  | **Accession Version** |
| **F4/80** | *CTGCACCTGTAAACGAGGCTT* | *GCAGACTGAGTTAGGACCACAA* | NM_010130 |
| **MCP1** | *TAAAAACCTGGATCGGAACCAAA* | *GCATTAGCTTCAGATTTACGGGT* | NM_011333 |
| **TNFα** | *CTGAACTTCGGGGTGATCGG* | *GGCTTGTCACTCGAATTTTGAGA* | NM_013693 |

F4/80: adhesion G protein-coupled receptor E1, also known as F4/80; MCP1: chemokine (C-C motif) ligand 2 (Ccl2), also known as MCP1; TNFα: tumor necrosis factor α.
